# Fires in rainforests: Quantifying litter bed flammability of cool temperate rainforests in eastern Australia

**DOI:** 10.1002/ajb2.70111

**Published:** 2025-10-14

**Authors:** Jamie E. Burton, Trent D. Penman, Ross J. Peacock

**Affiliations:** ^1^ FLARE Wildfire Research Group The University of Melbourne 4 Water Street Creswick 3363 VIC Australia; ^2^ New South Wales National Parks and Wildlife Service Locked Bag 5022 Parramatta 2124 NSW Australia; ^3^ Department of Natural Sciences Macquarie University NSW 2109 Australia

**Keywords:** Australia, climate change, eucalypt, fire regime, flammability, leaf traits, litter bed, rainforest, refugia

## Abstract

**Premise:**

Rainforests are usually too wet to burn, acting as natural barriers to fire spread and as refuges for biodiversity. However, climate change is increasing the risk of fire incursion into rainforests. Our capacity to manage these impacts is hindered by limited research on rainforest flammability.

**Methods:**

Fallen leaf and litter bed samples were collected from cool temperate rainforest and eucalypt forest in Willi Willi and Werrikimbe National Parks, New South Wales, Australia. Litter bed flammability of 13 common temperate rainforest species was measured in the laboratory. The flammability of rainforest litter beds and fire‐prone eucalypt forest litter beds were compared for 0.07 m^2^ and 7.2 m^2^ beds. We also examined links between flammability and key structural and chemical leaf traits.

**Results:**

Rainforest species varied in their litter bed flammability; 64% of the species had lower flammability than litter comprising eucalypt leaves, which typically occur in more fire‐prone environments. Species with smaller leaves and less leaf cellulose were associated with lower flammability. Rainforest litter beds had slower flame spread rates, smaller flames, and less material consumed compared to eucalypt litter beds.

**Conclusions:**

Fire characteristics in cool temperate rainforests will vary depending on the species composition of the litter bed, which depends on the litterfall composition of the rainforest stand. This study provides key insights into litter bed flammability in cool temperate rainforests in Australia, which will inform decisions on management of wildfires.

Australia is a flammable continent with many vegetation types that readily burn (Gill and Zylstra, 2005). Wildfire occurrence is controlled by four switches: (1) sufficient biomass (fuel), (2) availability to burn (dryness), (3) fire weather and (4) an ignition source (Bradstock, 2010; Nolan et al., 2020a). Wildfires occur when all these switches are “on”. In Australian temperate forests, fuel biomass is typically high and therefore not limiting to fire spread, except for the immediate period after fire or other disturbances (Raison et al., 1983). Therefore, fuel dryness is a key limitation on wildfire occurrence (Nolan et al., 2016). Different ecosystems have different moisture thresholds for fire occurrence (Duff et al., 2018). For example, moisture thresholds for wildfire are reached frequently in dry temperate eucalypt forest, leading to a regime of frequent (5‐ to 20‐year return intervals), moderate intensity (1000–5000 kW/m) shrub fires in spring and summer (Cheal, 2010; Murphy et al., 2013). In comparison, cool temperate rainforests in eastern Australia are typically too wet to burn and therefore experience infrequent (>50‐ to 100‐year return intervals), low‐intensity (<100 kW/m) surface fires in spring (Cheal, 2010; Murphy et al., 2013).

Cool temperate rainforests occur in patches that are often adjacent to or surrounded by fire‐prone eucalypt forest. A range of factors contribute to the low flammability of cool temperate rainforests in eastern Australia. First, cool temperate rainforests receive high rainfall and tend to occupy sheltered topographic positions such as polar‐facing slopes, creeks, and gullies (Bale et al., 1998; Wood et al., 2011; Leonard et al., 2014). As a result, cool temperate rainforests have dense, closed canopies that limit solar radiation input, promoting a humid, cool microclimate, leading to high fuel moisture contents relative to adjacent eucalypt forests (Crockett et al., 2006; Turton and Sexton, 2006; Styger, 2014). Second, cool temperate rainforests support higher decomposition rates resulting in low fuel biomass (Thomas et al., 2014). Third, cool temperate rainforests tend to have less grass and shrub cover (Baker et al., 2021), reducing the vertical spread of fire to the canopy. Last, cool temperate rainforest species may possess certain traits that lower their flammability. For example, leaves from Tasmanian cool temperate rainforest species generally have high ash contents and low volatile oil contents, meaning they propagate fire less readily compared to species in dry eucalypt forest (Dickinson and Kirkpatrick, 1985). Rainforest species may have higher live fuel moisture content (LFMC) in their leaves (Dickinson and Kirkpatrick, 1985); however, LFMC is a dynamic trait that varies depending on the plant traits (e.g., leaf size) and prevailing climatic conditions (Nolan et al., 2020b; Griebel et al., 2023). Rainforest species also may possess other traits, such as smaller leaves with less curl, which may reduce flammability via its effect on packing ratio (Burton et al., 2021).

In the last decade, several significant wildfires have impacted cool temperate rainforests in temperate Australia. This includes the 2019–2020 Black Summer fires (Collins et al., 2019; Nolan et al., 2020a; Fensham et al., 2024), where an estimated 11.2% (~2354 ha) of the cool temperate rainforest in New South Wales was burned (Peacock and Baker, 2022). The Black Summer fires were preceded by severe drought in much of eastern Australia (Nolan et al., 2020a). Drought is an important precursor to large and severe wildfire seasons in eastern Australia (Collins et al., 2022), and the frequency and severity of droughts are increasing under climate change (Abram et al., 2021). Drought reduces the fuel moisture differential between rainforests and adjacent fire‐prone eucalypt forest, increasing the probability of fire spread into rainforests (Collins et al., 2019). In addition, fire weather is intensifying during the fire season (Canadell et al., 2021; Richardson et al., 2021), increasing the potential for wildfires in eucalypt forest which then impact rainforests. Thus, two important switches for fire occurrence—the availability to burn (dryness) and fire weather—are changing as the climate changes, increasing the likelihood of fire impacting cool temperate rainforests.

Wildfires in rainforests present a significant challenge for both conservation and the management of landscape fire risk. Although many rainforest species can resprout post‐fire (Clarke et al., 2009; Trouve et al., 2021; Baker et al., 2022), the impacts of this changing fire regime on the condition and persistence of rainforest communities are unclear. Rainforests provide important biodiversity refuges during and after a wildfire (Leonard et al., 2014; Collins et al., 2019). Cool temperate rainforests also have high conservation value due to the presence of Gondwanan flora (e.g., Southern beeches, *Nothofagus* sp.) (Cavanaugh et al., 2010; Peacock and Baker, 2022) and the high level of endemism (Bale and Williams, 1993; Hunter, 2004; Adam, 2017), particularly of epiphytic and lithophytic lower plants (Franks and Bergstrom, 2000; Downing et al., 2014). The repeated encroachment of fire into rainforests may cause a myriad of impacts including increased rates of tree collapse, habitat loss and the gradual contraction of rainforest patches (Peacock and Baker, 2022; Bird et al., 2025).

To better manage the impacts of fire on cool temperate rainforests, we need to understand their flammability and underlying drivers. However, our knowledge of flammability in these ecosystems is limited as most fire behaviour research in Australia has focused predominately on drier forest types (Cruz et al., 2015). Most fires in cool temperate rainforests are surface fires; therefore, knowledge on rainforest flammability and fire behavior in the litter bed (or surface fuel) is critical for managing the impacts of fire on these important ecosystems and for managing landscape fire risk. Here, to better understand litter bed flammability in cool temperate rainforests of eastern Australia, we asked: How does litter bed flammability vary between common rainforest species? How do leaf traits influence litter bed flammability? How does flammability differ between rainforest litter beds and eucalypt litter beds? What are the implications for future fires in rainforest plant communities?

## MATERIALS AND METHODS

**Figure 1:**
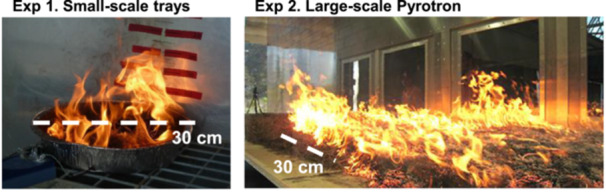
Overview of small‐scale circular tray experiments and large‐scale CSIRO Pyrotron experiments. Dashed line shows differing experimental scales. Image credits: left, R. Peacock; right, CSIRO Black Mountain.

To better understand litter bed flammability in cool temperate rainforests of eastern Australia, we set up flammability experiments at two scales in 2012 and 2013 with colleagues from the New South Wales Parks and Wildlife Service and CSIRO (Figure 1). In Experiment 1, small‐scale tests (0.07 m^2^) were done using small circular trays, a common approach in litter bed flammability research (Plucinski and Anderson, 2008; Grootemaat et al., 2017; Burton et al., 2021), with (1) single‐species leaf litter beds and (2) multispecies litter beds from rainforest and eucalypt forest (Figure 1, Exp. 1). For the large‐scale tests in Exp. 2, mixed litter beds were burned in a custom‐designed wind tunnel (7.2 m^2^, 1.5 × 4.8 m), known as the CSIRO Pyrotron (Sullivan et al., 2013) (Figure 1, Exp. 2). Further details on the study area, sample collection and flammability experiments are given below.

### Study area

**Figure 2:**
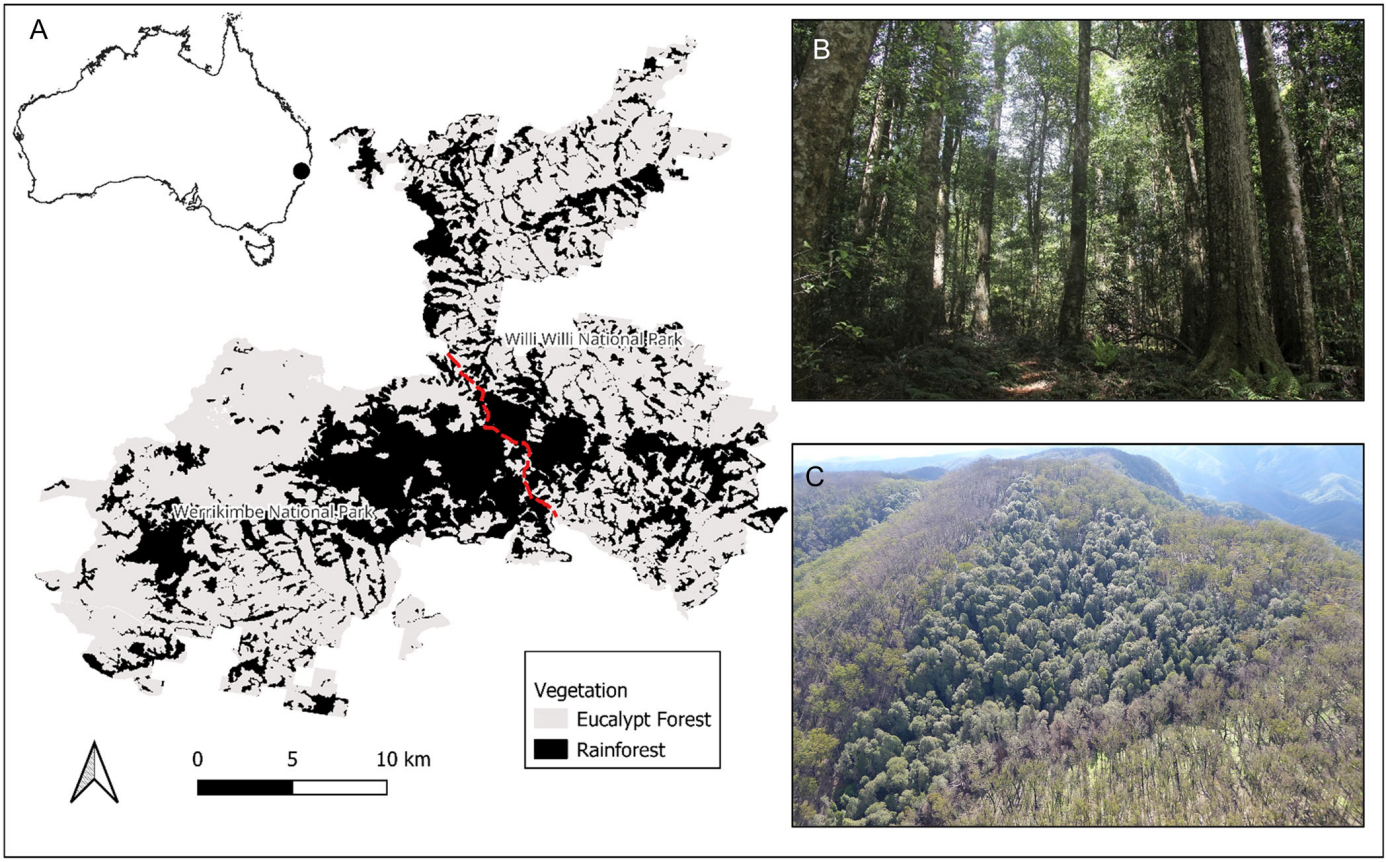
(A) Location of study area in eastern Australia, with extent of cool temperate rainforest (black) and eucalypt forest (grey) in the Willi Willi and Werrikimbe National Parks (red dashed line: boundary between the parks), (B) undisturbed *Nothofagus moorei* cool temperate rainforest and (C) View of a rainforest patch surrounded by burnt eucalypt forest, near Spokes Mountain, Werrikimbe National Park. In this patch, the surrounding vegetation (eucalypt forest) was burnt in the 2019/2020 Black Summer fire season, the fire scorched the perimeter of the rainforest patch and spread underneath the canopy burning the litter bed and understorey vegetation. Image credits: (B) R. Peacock, (C) NSW National Parks and Wildlife Service.

The study area is in eastern Australia in the Willi Willi and Werrikimbe National Parks (Figure 2A). The vegetation of focus is cool temperate rainforest, formally classed as *Nothofagus moorei–Ceratopetalum apetalum* sub‐alliance (Floyd, 1990) (Figure 2B). Cool temperate rainforest is located within a small altitudinal range (900–1240 m) and forms part of the Gondwanan Rainforests of Australia, a recognised UNESCO World Heritage Area property reserve. The reserve contains ancient plant communities that play a significant role in biodiversity conservation. The climate is cool temperate, with a mean annual rainfall of 1929 mm and a mean annual temperature of 14.6°C (1959–2024 average) (BOM Station 060068). Relative humidity usually exceeds 90%, and afternoon mists are common all year (Peacock and Baker, 2022).

*Nothofagus moorei* (F. Muell.) Krasser (Nothofagaceae) is the dominant canopy species of cool temperate rainforest, forming an open canopy (25–40 m tall) above a dense subcanopy (15–25 m tall) of *Ceratopetalum apetalum* D. Don, (Cunoniaceae), *Orites excelsus* R. Br. (Protecaeae), and *Doryphora sassafras* Endl. (Atherospermataceae) (Figure 2B). Other common species include *Ackama paniculosa* (F. Muell.) Heslewood (Cunoniaceae), *Callicoma serratifolia* Andrews (Cunoniaceae), *Cryptocarya foveolata* C.T. White & W.D. Francis (Lauraceae), *Cryptocarya meisneriana* F.M. Bailey (Lauraceae), *Elaeocarpus reticulatus* Sm. (Elaeocarpaceae), *Persoonia media* R. Br. (Proteaceae), *Quintinia verdonii* F. Muell. (Paracryphiaceae), *Schizomeria ovata* D. Don (Cunoniaceae), and *Trochocarpa laurina* (Rudge) R. Br. (Ericaceae).

Stand structures are of uneven or multiple ages, similar to the *Nothofagus cunninghamii* (Hook.) Oerst. (Nothofagaceae) forests of northwestern Tasmania (Hickey and Felton, 1991) and the Central Highlands of Victoria (Simkin and Baker, 2008). There is a continuous understorey cover of ground fern species including *Blechnum wattsii* Tindale (Blechnaceae) and *Sticherus lobatus* (R. Br.) Ching (Gleicheniaceae), and semiwoody vines such as *Ripogonum discolor* F. Muell. (Ripogonaceae).

**Figure 3:**
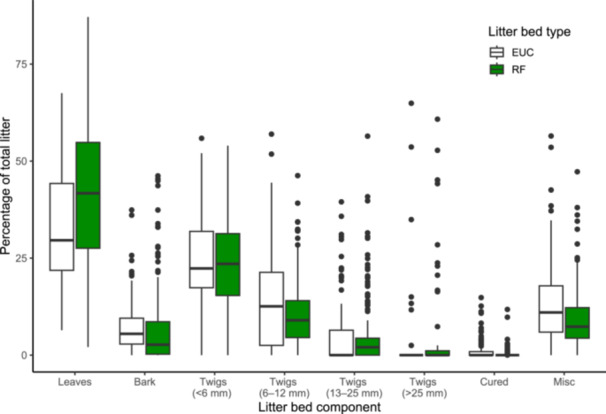
Litter bed composition between cool temperate rainforest (RF) and *Eucalyptus campanulata* open forest (EUC). Mean percentage contribution based on oven dry mass. Cured includes other dried material (e.g., grass, fern fragments). Misc. includes fragment material, remnants of capsules, petioles, etc. Colors represent litter bed types: rainforest (green) and eucalypt (white). Box plots show median (horizontal line within box), 25th and 75th percentile (box boundaries), highest and lowest values (whiskers), outliers (filled circle outside box). For details on how litter composition was measured see section Measurement of litter components in Appendix S1.

The litter bed of cool temperate rainforest is generally sparse with a low litter load (mean ± 1 SD, 11.33 ± 6.01 t/ha). Adjacent tall open eucalypt forests dominated by *Eucalyptus campanulata* R.T. Baker & H.G.Sm [Myrtaceae] typically have higher litter loads (14.53 ± 6.21 t/ha; Peacock and Baker, 2022). Cool temperate rainforest has limited grass and shrub fuels, no ladder fuels (dead or live vegetation, such as tall grasses, shrubs, that connect the forest floor to the tree canopy, allowing a surface fire to easily spread into the canopy), high litter turnover, and low input rates relative to adjacent eucalypt forests (Peacock and Baker, 2022). Total biomass and leaf litter fall peaks in spring, between September and November (Vogado et al., 2024). Leaves make up a significant proportion of cool temperate rainforest litter beds (42%), followed by fine (<6 mm) twigs (24%) (Figure 3).

Wildfires in cool temperate rainforest are generally rare but when they do occur, they tend to be in late spring (November) and are typically surface fires confined to the litter fuel (Peacock and Baker, 2022) (Figure 2C). Wildfires typically follow a period of drought, as this causes soil volumetric moisture content to fall below 10–20% and the litter bed to dry out throughout the profile (Peacock and Baker, 2022). Deep drying of the litter profile was a key contributing factor to the extensive wildfires in Victoria in 2009 which saw fire spread into fire refugia, such as southern slopes and gullies, which contains rainforest vegetation (Cawson et al., 2020). In Tasmania, *Nothofagus* rainforest is only able to sustain fire at times of the year when the total rainfall in the preceding month has been less than 50 mm (Styger, 2014; Styger and Kirkpatrick, 2015). Data on fire frequency in cool temperate rainforests in northern New South Wales is sparse; however, mapping and management records dating from the 1950s indicate an average interval of one to two fires per century in the Willi Willi and Werrikimbe National Parks region (Peacock and Baker, 2022).

### Sample collection

Fully expanded leaves from canopy or subcanopy rainforest species were collected in February 2013 from Willi Willi National Park (latitude –31°09′53.27″, longitude 152°23′06.40″ 1030 m a.s.l.) for laboratory trait analysis. Species were selected based on their role as canopy or subcanopy dominants that have significant contribution to litterfall and standing litter biomass. Fourteen species were selected including one non‐rainforest species, *Eucalyptus campanulata*, common in adjacent eucalypt forests (Table 1). Collection of material was aided by an east coast low weather event (22–24 February 2013) where 380 mm of rain fell in 3 days accompanied by high wind gusts leading to widespread tree falls providing ready access to fresh canopy branches at ground level. *Orites excelsus* has multiple leaf forms reflecting growth stage, so we only sampled the mature multilobed leaf form to standardize leaf properties. Approximately 700 fully expanded leaves per species were sampled from among five trees for leaf trait determination and single‐species litter bed flammability experiments.

**Table 1:** List of species collected for single‐species leaf litter bed experiments.

Species	Abbreviation	Common name	Growth form	Family
*Ackama paniculosa*	Calpan	Soft corkwood	Medium tree	Cunoniaceae
*Callicoma serratifolia*	Calser	Black wattle	Tall shrub or small tree	Cunoniaceae
*Ceratopetalum apetalum* a	Cerape	Coachwood	Medium tree	Cunoniaceae
*Cryptocarya foveolata* a	Cryfov	Mountain walnut	Medium to large tree	Lauraceae
*Cryptocarya meisneriana* a	Crymei	Thick‐leaved laurel	Shrub or small tree	Lauraceae
*Doryphora sassafras* a	Dorsas	Sassafras	Medium tree	Atherospermataceae
*Elaeocarpus reticulatus*	Elaret	Blueberry Ash	Shrub or small tree	Elaeocarpaceae
*Eucalyptus campanulata* a	Euccam	New England blackbutt	Large tree	Myrtaceae
*Nothofagus moorei* (F. Muell.)^a^	Notmoo	Antarctic beech	Large tree	Nothofagaceae
*Orites excelsus* a	Oriexc	Mountain silk oak	Medium to large tree	Proteaceae
*Persoonia media* a	Permed	Geebung	Erect to spreading shrub to tree	Proteaceae
*Quintinia verdonii*	Quiver	Grey possumwood	Medium tree	Paracryphiaceae
*Schizomeria ovata*	Schova	Crabapple	Medium to large tree	Cunoniaceae
*Trochocarpa laurina*	Trolau	Tree heath	Compact shrub to crooked tree	Ericaceae

^a^ Species for which both morphological and chemical leaf traits were measured.

Mixed litter beds were collected from sites in Willi Willi and Werrikimbe National Parks across latitude –31°09′53.27″, longitude 152°23′06.40″ (1030 m a.s.l.) and latitude –31°08′49.84″, longitude 152°21′40.99″ (1040 m a.s.l.). Mixed litter beds were collected in the field using a 35‐cm‐diameter, steel sampling ring between 7 and 13 March 2013. All fine material (leaves, bark and twigs of <6 mm thickness) was collected down to mineral earth.

Additional mixed litter bed samples were collected for testing in the Pyrotron. Samples were collected within Willi Willi and Werrikimbe National Parks, northern New South Wales between 16 and 18 October 2012. Sampling locations were dispersed between latitude –31°09′23.39″, longitude 152°22′13.16″ 1100 m a.s.l. and latitude –31°08′52.64″, longitude 152°21′42.46″ 1040 m a.s.l. Sampling locations were randomized within a tall open forest of *Eucalyptus campanulata* and a cool temperate rainforest, and the ecotone between the two. The ecotone vegetation is composed of a mixture of rainforest and eucalypt species and occurs along the margins of both vegetation types. Each sample consisted of all the fine (<6 mm thickness) litter (to the mineral earth) within a 1.5 × 4.8 m sample area. Litter depth was measured at 1 m increments with a litter depth gauge.

### Leaf trait measurements

Fresh mass was measured in the field within 1 h of field collection with a balance accurate to 0.001 g. Fresh leaves were stored in a field refrigerator at 4–7°C in sealed plastic bags before transport to the laboratory for leaf trait determination. Leaf morphology was measured for all 14 species, using approximately 150 leaves per species. Leaf area (one‐sided, cm^2^) was measured with a LICOR 3100 C area meter (LICOR, Lincoln, NE, USA). Specific leaf area (SLA, cm^2^/g) was calculated as one‐sided leaf area divided by leaf dry mass. Leaf dry mass was measured after oven‐drying at 70°C for 48 h based on the protocol recommended by Cornelissen et al. (2003).

For leaf chemistry, phosphorous content, total carbon content, nitrogen content, cellulose content, lignin content, total Acid Detergent Fiber (ADF, cellulose and lignin), insoluble ash content and soluble cell content were analyzed for eight of the 14 species. Five adult leaves from a species were bulked, oven‐dried at 70°C for 48 h, then ground to a powder with a mortar and pestle (except *Eucalyptus campanulata* = 2 leaves, *Cryptocarya foveolata* = 1 leaf). All the analyses were done by the Analytical Services Laboratory (University of Queensland, Queensland, Australia). Phosphorous content (mg/kg) was measured using nitric acid digestion and inductively coupled plasma‐optical emission spectroscopy (ICP‐OES). Total carbon and total nitrogen (% dry mass) were measured by combustion and infrared detection using a LECO TruSpec CHN automated analyzer (LECO Corporation, St. Joseph, Michigan, USA). Cellulose, lignin, insoluble ash and soluble fractions were determined by sequential ADF extraction. The method was an adaptation of Van Soest procedure (Goering and Van Soest, 1970) with variants for Ash Content, Lignin and Cellulose determination.

### Flammability experiments

Two flammability experiments were done: (1) a small‐scale circular tray experiment and (2) large‐scale experiment in a Pyrotron (Figure 1). The small‐scale experiments, using single‐species or mixed‐species leaf litter beds, were done in a wind‐proof chamber in a semi‐enclosed shed at the Port Macquarie New South Wales National Park Service Depot using the procedure of Plucinski and Anderson (2008). For the single‐species beds, two replicates per species were tested. For the mixed‐litter beds, 48 rainforest and 40 eucalypt litter beds were tested. All samples were oven dried at 70°C for 48 h before burning. A subsample (6–10 g dry mass) of the oven‐dried litter was used for gravimetric moisture determination. Litter was reconstructed into a circular tray (30 cm diameter, 0.07 m^2^) with a standardized mass of 50 g for all samples and ignited manually by lighting a cotton ball placed in the center of the tray with a handheld gas lighter. A video camera recorded the experiment. During the experiments, ambient temperature ranged from 19.0 to 29.0°C (mean ± 1 SD, 23.5 ± 2.3), relative humidity ranged from 53 to 91% (63 ± 11.2%), and dead fine fuel moisture content ranged from 4.8 to 15.2% (9.01 ± 2.4%). The following flammability metrics were measured: time to ignition (s), time taken for sample to ignite after exposure to the ignition source; flaming duration (s), time from ignition to cessation of flaming; flame spread rate (cm/s), distance to the edge of the tray (12.5 cm) divided by the time taken for flames to reach the edge of the tray; maximum flame height (cm), measured using the video footage and graduated scale; maximum temperature (°C), reached during the burn; and consumption (% mass loss), proportion of sample consumed during experiment, calculated from pre‐ and post‐burn mass. Temperature was measured using three thermocouples (Type K M1 250 mm; Labfacility, Bognor Regis, UK) spaced 120° apart on the surface of the litter. A Pico Thermocouple USB TC‐O8 (PICO Technology, St Neots, UK) data logger recorded temperature every 100th of a second.

The large‐scale experimental burns were done in the CSIRO Pyrotron combustion wind tunnel located in Canberra, Australia (Sullivan et al., 2013) (Figure 1). The Pyrotron allows larger litter bed reconstruction compared to the circular tray method (7.2 m^2^ vs. 0.07 m^2^) and provides information on the likelihood of sustained fire spread. Two replicates per litter bed type (rainforest, ecotone, and eucalypt) were tested. Litter beds were oven‐dried at 70°C for 48 h to fuel moisture contents close to 10%. Litter beds were reconstructed in the working section of the Pyrotron (1.5 × 4.8 m, 7.2 m^2^) to match field conditions and the dimensions of the field sampling unit. Mean oven dry fuel mass of rainforest, ecotone and eucalypt in the field was 9.82 t/ha, 11.25 t/ha and 13.63 t/ha, respectively. Depth of the litter bed depth in five locations was measured before ignition (mean litter bed depth: rainforest = 11.6 mm, ecotone = 14.5 mm and eucalypt 14.3 mm) to ensure they were similar to the field litter depth for each litter bed type. For fine fuel moisture content, a small sample was also removed from five locations before the ignition for gravimetric moisture determination. For each experiment, samples were ignited with a 1.5‐m ignition line and wind speed was set at 1.5 m/s (5.4 km/h). The ambient temperature ranged from 21.7 to 31.7°C (mean ± 1 SD, 26.7 ± 3.7), relative humidity ranged from 23.8 to 37.2% (30.5 ± 4.9%) and dead fine fuel moisture content ranged from 6.7 to 10% (7.9 ± 1.2%) during testing. Samples were burnt over 2 days in summer 2012 (December 11 and 12). Flame height was estimated visually every 0.5 m using a graduated scale on the Pyrotron wall. The time of flame arrival was visually estimated at increments of 0.5‐ to 4 m to quantify flame spread rate.

### Data analyses

We first examined whether ambient air temperature, relative humidity and fuel moisture content influenced the results of the circular tray experiments (single‐species, mixed‐species litter beds). We found no significant relationships between these covariates and the flammability metrics (Appendix S2: Figure S1). However, both replicates of *Trochocarpa laurina* and one replicate of *Ackama paniculosa* had high moisture contents near 15%.

To understand how litter bed flammability varies between common rainforest species, we compared mean values for the flammability metrics (Appendix S2: Table S2). Because only two replicates per species were measured for litter bed flammability, raw data were plotted and the mean value displayed.

Next, we examined how leaf traits influence litter bed flammability using the mean values of the flammability metrics and leaf traits for each species (Appendix S2: Tables S1, S2). We quantified the correlation between leaf traits and litter bed flammability using Spearman's rank correlation coefficient (*ρ*) due to the non‐normality of some variables (consumption, time to ignition, flame spread, and maximum temperature). Strong correlations between traits and flammability (rho ≥ ±0.70) were then explored using bivariate regression (Appendix S2: Table S3). Linear models were used for time to ignition and maximum temperature and logistic models were used for consumption. Linear models were fit using the function lm, and logistic models were fit using the function glm’, with a binomial distribution, in R version 4.3.3 (R Core Team, 2022). Models were evaluated using a measure of model fit (adjusted *R* ^2^ for linear models and McFadden's pseudo *R* ^2^ for logistic models) and error (root mean square error, RMSE, for linear models only). A higher *R* ^2^ and lower RMSE indicates better model fit. The function PseudoR2 from the R package DescTools version 0.99.23 was used to calculate McFadden's pseudo *R* ^2^ (Signorell, 2017).

For the mixed species litter bed results, mean values of flammability metrics were calculated for eucalypt and rainforest litter beds. We used box plots to examine differences in flammability metrics between rainforest and eucalypt litter beds. Flammability between rainforest and eucalypt litter beds were tested for differences using paired *t*‐tests after data were tested for normality using the Shapiro–Wilk test. If differences were not normal, then Wilcox rank sum test was used to test for differences in flammability between rainforest and eucalypt litter beds. For the Pyrotron experiment, flame spread was plotted as a function of time. Flame height during the experiment was compared between litter bed types using paired t‐tests. All data analysis was performed in R version 4.3.3 (R Core Team, 2022). Normality was assessed using the function ‘shapiro.test’ function in the ‘stats’ package and significance testing was done with the functions ‘t.test’ or ‘wilcox.test’, both from the ‘stats’ package in R.

## RESULTS

### Litter bed flammability of rainforest species

**Figure 4:**
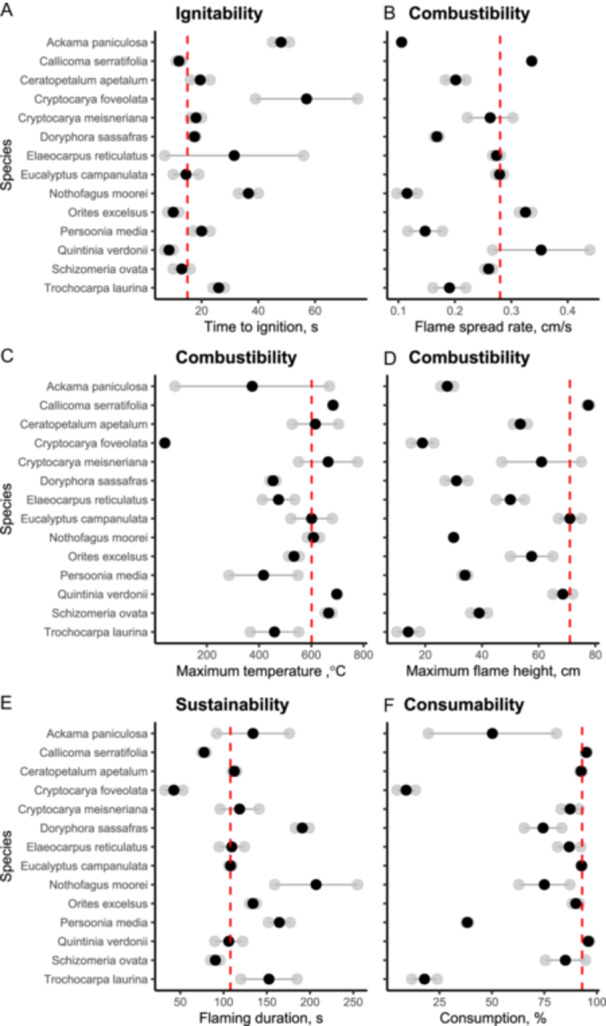
Flammability measurements from the small‐scale circular tray experiment using single‐species leaf litter beds. (A) Ignitability (time to ignition). (B–D) Combustibility: (B) flame spread rate, (C) maximum temperature, (D) maximum flame height. (E) Sustainability (flaming duration). (F) Consumability (consumption). Grey dots: raw data; black dot: mean. Red dashed line: is the mean value for *Eucalyptus campanulata*. Species are in alphabetical order. Note: None of the *Cryptocarya foveolata* replicates sustained burning, so they had low mean maximum temperatures.

*Cryptocarya foveolata* was the least flammable at the litter bed scale, with none of the replicate burns spreading to the edge of the tray (~15 cm) (Figure 4). It also had a long ignition time (57 s), low maximum temperatures (38.9°C), little amount of material consumed (9.1%) and low flame height (19 cm) (Figure 4). The low maximum temperature reached is reflected in the low proportion of material consumed. Other species with low flammability were *Ceratopetalum apetalum*, *Nothofagus moorei*, *Cryptocarya foveolata*, *Trochocarpa laurina*, *Ackama paniculosa*, and *Persoonia media* (Figure 4). Species with higher flammability at the litter bed‐scale included *Quintinia verdonii*, *Callicoma serratifolia*, and *Eucalyptus campanulata* (Figure 4). All these species had short ignition times (9–15 s), fast flame spread rates (0.28–0.35 cm/s), and tall flames (69–78 cm) (Figure 4).

### Leaf traits and litter bed flammability

**Figure 5:**
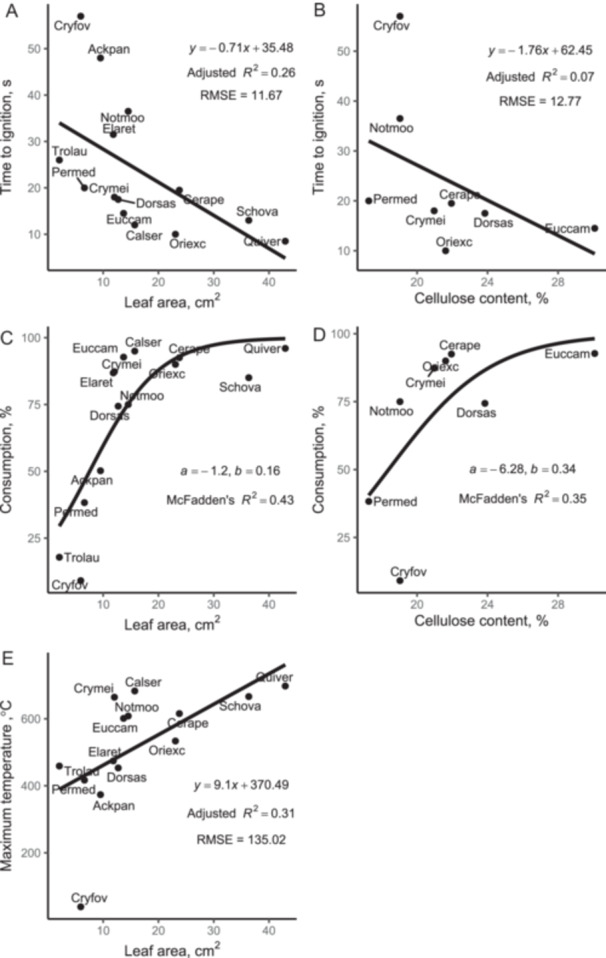
Relationship between litter bed flammability and leaf traits. Dots are mean values for species; abbreviations are defined in Table 1. Linear regressions shown as solid black lines in panels A, B, and E; logistic regression (binomial distribution) is in panels C and D. Equation, model fit (Adjusted *R* ^2^, McFadden's Pseudo *R* ^2^ and error (Root Mean Square Error [RMSE]) are shown in each panel. For the logistic regression, the equation coefficients *a* and *b* are shown ($\text{Consumption}\;=\;\frac{1}{1\;+\;e^{-(a+\mathrm{bx})}}$).

Both structural and chemical leaf traits were strongly correlated with some litter bed flammability metrics (Figure 5; Appendix S2: Table S3). Leaf area was positively correlated with consumption (McFadden's *R* ^2^ = 0.43, *ρ* = 0.79, *P* < 0.0007) and maximum temperature (Adjusted *R* ^2^ = 0.31, *ρ* = 0.81, P = 0.0004) and negatively correlated with time to ignition (Adjusted *R* ^2^ = 0.26, *ρ* = –0.75, P = 0.002). Cellulose content was negatively correlated with time to ignition (Adjusted *R* ^2^ = 0.07, *ρ* = –0.70, P = 0.04) and positively correlated with consumption (McFadden's Pseudo *R* ^2^ = 0.35, *ρ* = 0.71, P = 0.05).

### Flammability of rainforest mixed litter beds

**Figure 6:**
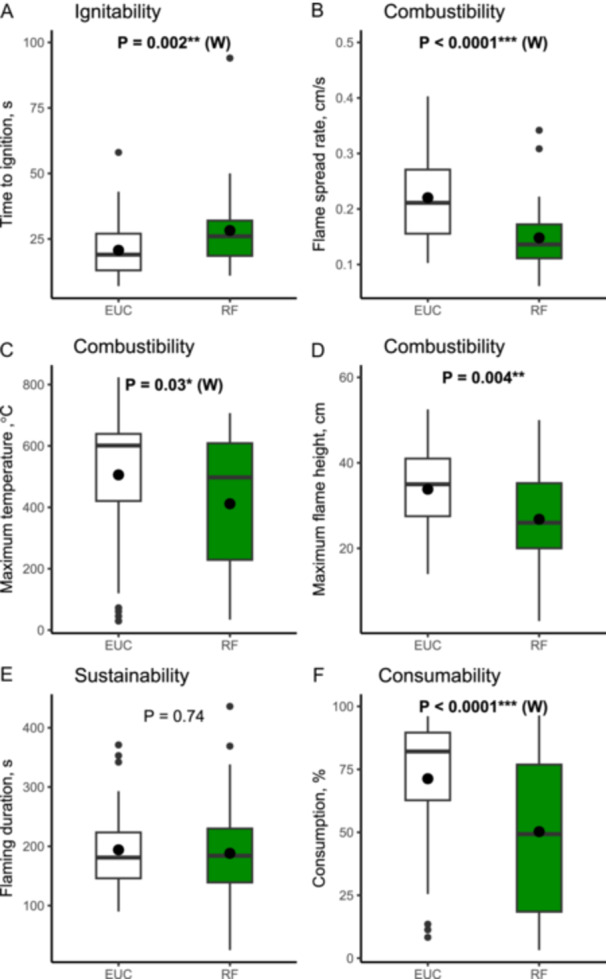
Flammability measurements from the small‐scale circular tray experiment using mixed litter beds. (A) Ignitability (time to ignition). (B–D) Combustibility: (B) flame spread rate, (C) maximum temperature, (D) maximum flame height. (E) Sustainability (flaming duration). (F) Consumability (consumption). Colors represent litter bed types: rainforest (RF, green) and eucalypt (EUC, white). Box plots show median (horizontal line within box), mean (black filled circle within box), 25th and 75th percentile (box boundaries), highest and lowest values (whiskers) and outliers (filled circle outside box). Significant differences are in bold based on paired *t*‐test or Wilcox rank sum test (indicated by W): *P ≤ 0.05, **P ≤ 0.01, ***P ≤ 0.001.

Rainforest litter beds took longer to ignite (mean rainforest vs. eucalypt, 28.2 vs. 20.7 s, *P* = 0.002), slower flame spread rates (0.15 vs. 0.22 cm/s, *P* < 0.0001), lower flame heights (27 vs. 34 cm, *P* = 0.004), lower maximum temperatures (412 vs. 506°C, *P* = 0.03) and less material consumed (50.3 vs. 71.3%, *P* < 0.0001) (Figure 6). There was no significant difference in flaming duration (188 vs. 194 s, *P* = 0.74) (Figure 6).

**Figure 7:**
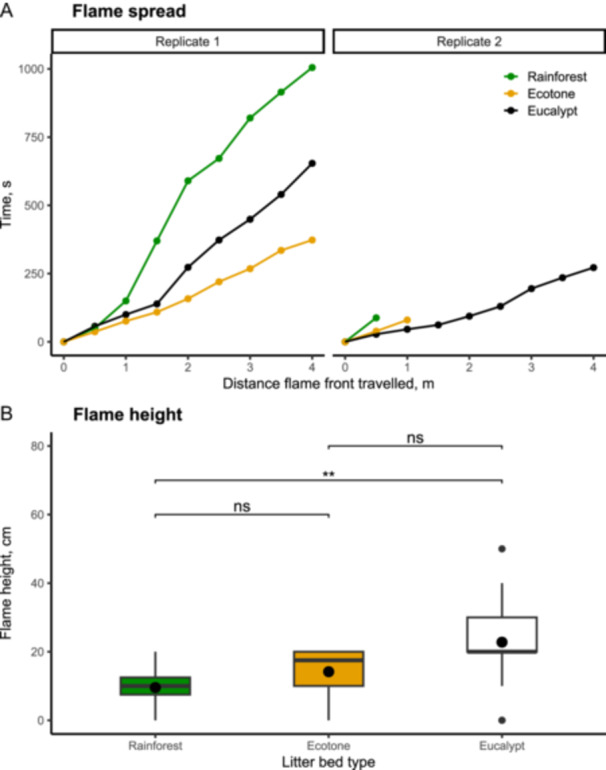
Flammability measurements from the large‐scale CSIRO Pyrotron experiments using mixed litter beds. (A) Flame spread rate in litter beds from rainforest, ecotone, and eucalypt forest. (B) Flame height. Box plots show median (horizontal line within box), mean (black filled circle within box), 25th‐ and 75th‐percentile (box boundaries) and highest and lowest values (whiskers) and outliers (filled circle outside box). Significance values determined using t‐test: *P ≤ 0.05, **P ≤ 0.01, ***P ≤ 0.001, ns = not significant.

The results of the large‐scale Pyrotron experiments (Figure 7) were similar to those of the small‐scale circular tray experiments (Figure 6). Rainforest litter beds had lower flame spread rates compared to ecotone and eucalypt litter beds (Figure 7A). The second replicate in rainforest and ecotone litter beds did not sustain combustion, despite having dry litter beds (fuel moisture contents: 7.5% and 8.5%, respectively). The second replicate in eucalypt litter bed had the fastest flame spread rate and shortest flaming duration. Rainforest litter beds had significantly lower flame heights compared to eucalypt forest litter beds (mean rainforest vs. eucalypt, 10 cm vs. 23 cm, *P* = 0.003) (Figure 7B). There was no significant difference in flame height between ecotone and rainforest (14 cm vs. 10 cm, *P* = 0.12) nor ecotone and eucalypt forest (14 cm vs. 23 cm, *P* = 0.06) (Figure 7B).

## DISCUSSION

### Litter flammability of cool temperate rainforest species

Cool temperate rainforest species varied in their litter bed flammability. Litter beds from most (64%) rainforest species were less flammable than *Eucalyptus campanulata*, a common canopy tree in adjacent tall open eucalypt forests. Species with low flammability included *Ceratopetalum apetalum*, and *Nothofagus moorei*. However, three species (*Quintinia verdonii*, *Orites excelsus*, and *Callicoma serratifolia*) had a flame spread rate similar to that of *Eucalyptus campanulata*. Two leaf traits, leaf area and cellulose content, were strong drivers of flammability, and we discuss the mechanisms below.

Litter beds composed of small‐leaved cool temperate rainforest species took longer to ignite, released less heat (lower maximum temperatures) and had less material consumed. Small‐leaved species which exhibited low flammability were *Cryptocarya foveolata, Ceratopetalum apetalum, Nothofagus moorei*, *Trochocarpa laurina, Ackama paniculosa*, and *Persoonia media*. *Cryptocarya foveolata* was distinctly non‐flammable, with the litter beds failing to ignite despite the low moisture content (8%). Although the packing ratio was not quantified in this study, it is likely these species form denser litter beds (higher packing ratio) because smaller leaves pack more densely (Burton et al., 2021). A higher packing ratio means less oxygen is available for combustion, making litter beds less flammable (Scarff and Westoby, 2006; Burton et al., 2021).

Litter beds made up of species with cellulose‐rich leaves were quicker to ignite and had more material consumed. Our results align with those of Wei et al. (2025) who found cellulose was a major driver of time to ignition and mass loss in subtropical leaf litter beds, with shorter time to ignition and greater mass loss in litter beds made‐up of leaves high in cellulose. The pyrolysis of cellulose results in by‐products such as phenols and other compounds with high calorific value (Younes et al., 2024). Thus, leaves that have more cellulose may produce more gases that when coupled with abundant oxygen can favor flame propagation, leading to shorter ignition times and greater consumption (Scarff and Westoby, 2006; Younes et al., 2024). Unfortunately, chemical leaf traits were only measured for 50% of the species in the study, so our results need to be interpreted with caution. Moreover, only selected chemical traits were measured, so the importance of other traits (e.g., terpenes, tannins) cannot be ruled out (Ormeño et al., 2009; Romero et al., 2019). It is also difficult to disentangle the relative importance of chemical vs. structural leaf traits based on our data set. Future studies are needed to examine the roles of chemical vs. structural leaf traits in determining litter bed flammability across a wider range of plant species.

At the community scale, we found rainforest litter beds were less flammable than eucalypt litter beds. Rainforest litter beds (when dry) can burn but generally take longer to ignite, spread more slowly and have smaller flames compared to eucalypt litter beds. Moreover, fires are less intense (lower maximum temperatures), and less material is consumed, leaving more unburned material, compared to eucalypt litter beds. The lower flammability was observed at both the smaller scale (circular trays) and at the larger scale (Pyrotron). In the Pyrotron experiments, despite the litter being dry (8.5% ecotone, 7.5% rainforest), fire only spread in one of two replicates for both the rainforest and the ecotone samples. We suggest that the slower flame spread relates to (1) rainforest litter beds having less litter that is more sparsely dispersed litter and (2) differences in species composition. Both *Ceratopetalum apetalum* and *Nothofagus moorei* are dominant contributors to leaf fall in cool temperate rainforests (Vogado et al., 2024) and so make‐up a substantial proportion (78%) of the leaf component of rainforest litter beds (35.2 and 43.7%, respectively; R. Peacock, New South Wales National Parks and Wildlife Service, Parramatta NSW Australia, personal communication). Thus, the lower flammability of *Ceratopetalum apetalum* and *Nothofagus moorei* leaf litter beds at the species‐level may impact the litter flammability at the community scale (i.e., in the mixed litter beds). Further controlled tests using known proportions of flammable and non‐flammable litter are required to better understand the dampening effect of these species (de Magalhães and Schwilk, 2012; Della Rocca et al., 2018; Zhao et al., 2019; Wei et al., 2025). In addition, other factors may be at play, such as component composition (e.g., proportion of fine twigs) and bulk density (Ganteaume et al., 2011; Burton et al., 2023). The proportion of fine (<6 mm) twigs may be important as they make up, on average, 25% of the litter in rainforests (Figure 3) and can promote or dampen fire behavior, depending on species traits such as density and structure (Zhao et al., 2019). Further work is needed to determine how these different litter bed attributes influence flammability in cool temperate rainforests.

Interestingly, our findings are in contrast with those of Clarke et al. (2014), who found no significant difference in litter bed flammability between rainforest and eucalypt‐dominated vegetation. This discrepancy may stem from variations in sample size for the circular tray experiments (3 in the study by Clarke et al. [2014] vs. 40 in our study) or differences in the specific composition and structure of the litter beds tested because Clarke et al. (2014) focused on warm temperate rainforests, whereas our study examined cool temperate rainforests. Another important consideration is that all flammability tests in our study were performed using oven dried litter samples, with a mean moisture content of 9% (from 4.8 to 15.2%). Litter beds from *Trochocarpa laurina* and *Ackama paniculosa* were at the upper end of this range, with moisture contents of 15.2 and 15.1%, respectively. While we did not detect a significant effect of moisture content on overall flammability results, we cannot resolve whether the low flammability observed in these two species is the result of leaf traits or moisture content, or a combination of the two. Nonetheless, understanding moisture thresholds in ignitability and how this is influenced by species composition and litter bed structure, is an important next step (Styger, 2014; Kauf et al., 2018, 2019; Burton et al., 2023) and will provide insight into the moisture thresholds required for ignition, assisting fire management planning and response.

### Implications for future fires

Climate change poses a significant threat to cool temperate rainforest (ANU, 2009; Narsey et al., 2020). More‐frequent warmer, drier conditions could reduce the moisture differential between rainforest and adjacent eucalypt forest, increasing the chance of fire incursion into rainforest (Collins et al., 2022; Cawson et al., 2024; Gordon et al., 2025), a phenomenon described as fire creeping into long‐term fire refugia (Worth et al., 2016). The impact of wildfire on rainforests is likely to be most relevant at rainforest margins, due to the incursion of fire from more flammable vegetation rather than ignitions starting in the rainforests themselves. Whilst rainforest litter beds are less flammable than adjacent fire‐prone eucalypt litter beds, if conditions are sufficiently dry, they can burn, albeit at lower intensity. Overall, it can be expected that surface fires in rainforests will be slower, release less heat and generate shorter flames compared to adjacent fire‐prone eucalypt forest. However, fire behavior in rainforests will vary depending on the overstorey species composition and the subsequent species composition in the litter bed. Fires may not spread or be less intense (smaller flames, lower maximum temperatures) if litter beds are dominated by non‐flammable species (e.g., *Cryptocarya foveolata*) that have small leaves and low cellulose content. At the other end of the spectrum, fires may be more intense and have faster spread if litter beds are dominated by more flammable species (e.g., *Quintinia verdonii*) that have larger leaves.

The regeneration of plant species post‐wildfire on the rainforest margins also has implications for future fires (Fletcher et al., 2020). For example, *Callicoma serratifolia* is a common pioneer after a wildfire, having been observed frequently recolonizing rainforest margins (Crockett et al., 2006; Peacock and Baker, 2022; Benwell and Williams, 2024). The presence of *Callicoma serratifolia* on rainforest margins may increase the likelihood of future fire due to its flammable litter beds. With repeated fires favouring the regeneration of *Callicoma serratifolia*, there may be continuing contraction of rainforest patches and a promotion of a more flammable species composition at rainforest margins.

## CONCLUSIONS

In this study, we assessed the flammability of cool temperate rainforest litter beds. We found that rainforest species vary in their litter bed flammability, primarily driven by variation in leaf size and cellulose content. We show that, under dry conditions, surface fires in rainforests can occur but they will be slower, release less heat and consume less material compared to adjacent fire‐prone eucalypt forest. However, fires characteristics (e.g., intensity, flame spread rate, time to ignition, amount consumed) may vary depending on the overstorey species composition, and the subsequent species composition in the litter bed. Our findings contribute to a greater understanding of surface fire behavior in rainforest communities and provide important insight for management of these high conservation value ecosystems as climate changes.

## AUTHOR CONTRIBUTIONS

R.J.P. designed the study and collected the data. J.E.B. analyzed the data and wrote the manuscript. All authors contributed to writing and editing.

## Supporting information


**Appendix S1.** Measurement of litter components.

**Appendix S2.** Correlation plot among flammability metrics and ambient conditions, table of mean leaf trait values, table of mean litter flammability and correlations between leaf traits and litter flammability.

## Data Availability

All data sets generated and/or analyzed during the current study have been deposited at Figshare: https://figshare.com/s/8652ff00d6100b21fa46.
