# Supplementary material for: Fires in rainforests: Quantifying litter bed flammability of cool temperate rainforests in eastern Australia
Source: Am J Bot. 2025 Oct 14;112(10):e70111. doi: 10.1002/ajb2.70111 (PMC12572681; doi:10.1002/ajb2.70111)
Supplement: Supplementary file 1 — Appendix S1. Measurement of litter components. [file AJB2-112-e70111-s002.docx]

**Appendix S1.** Measurement of litter components.

Fine fuels were sampled across Werrikimbe and Willi Willi National Parks in northern NSW between April 2010 and September 2013. Sampling was stratified by vegetation formations, specifically cool temperate rainforest and northern hinterland wet sclerophyll forests (Keith 2004), using vegetation mapping and locally validated diagnostic species lists. Sampling locations ranged between latitude 152.23’10.08 and longitude -31.9’49.883 and latitude 152.21’4.236 and longitude -31.7’2.109. Cool temperate rainforest sites had remained unburnt for at least 50 years, while the northern hinterland wet sclerophyll forest sites had been unburnt for 26–29 years. A total of 106 samples were collected from northern hinterland wet sclerophyll forests and 211 from cool temperate rainforest.

A steel litter ring with an area of 0.096 m² and a sharpened cutting edge was used to sample fine fuels, severing twigs and grasses. This method, in use since the 1960s, ensures standardized data collection (van Loon, 1969). Litter depth was also measured before collection to enable calculation of bulk density (total dry mass of fuel / litter depth x sample area, g/cm^3^). All fine litter material (leaves, twigs and bark) up to 25 mm in thickness was collected from the forest floor, with soil and stone material removed in the laboratory. The 25-mm threshold has been conventionally used since the 1960s because twigs up to this size are considered "available" fuel, particularly under conditions during the fire season (van Loon, 1969; Bridges, 2004). This practice was adopted by forest agencies as part of fire effects research initiated in the mid-1960s.

In the laboratory, samples were dried at 70°C for 48 h to a constant mass, after which they were sorted into eight components following van Loon (1969):

- Twigs < 6 mm diameter
- Twigs 6 to < 13 mm diameter
- Twigs 13 to < 25 mm diameter
- Twigs > 25 mm diameter (excluded from total dry weight calculations)
- Bark
- Leaves
- Cured vegetation
- Miscellaneous organic material (including charcoal, eucalypt fruits, and floral fragments)

*References*

Bridges, RG (2004). Fine fuel in the dry sclerophyll forests of south-eastern New South Wales. Australian Forestry, 67(2), 88–100. https://doi.org/10.1080/00049158.2004.10676212

Keith DA (2004) Ocean shores to desert dunes: the native vegetation of New South Wales and the ACT. Department of Environment and Conservation, Sydney.

Van Loon, AP (1969) Investigations into the effects of prescribed burning of young, even-aged Blackbutt. Forestry Commission of NSW Research Note No. 23, 49 pp.
