# Supplementary material for: Fires in rainforests: Quantifying litter bed flammability of cool temperate rainforests in eastern Australia
Source: Am J Bot. 2025 Oct 14;112(10):e70111. doi: 10.1002/ajb2.70111 (PMC12572681; doi:10.1002/ajb2.70111)
Supplement: Supplementary file 2 — Appendix S2. Correlation plot among flammability metrics and ambient conditions, table of mean leaf trait values, table of mean litter flammability and correlations between leaf traits and litter flammability. [file AJB2-112-e70111-s001.docx]

**
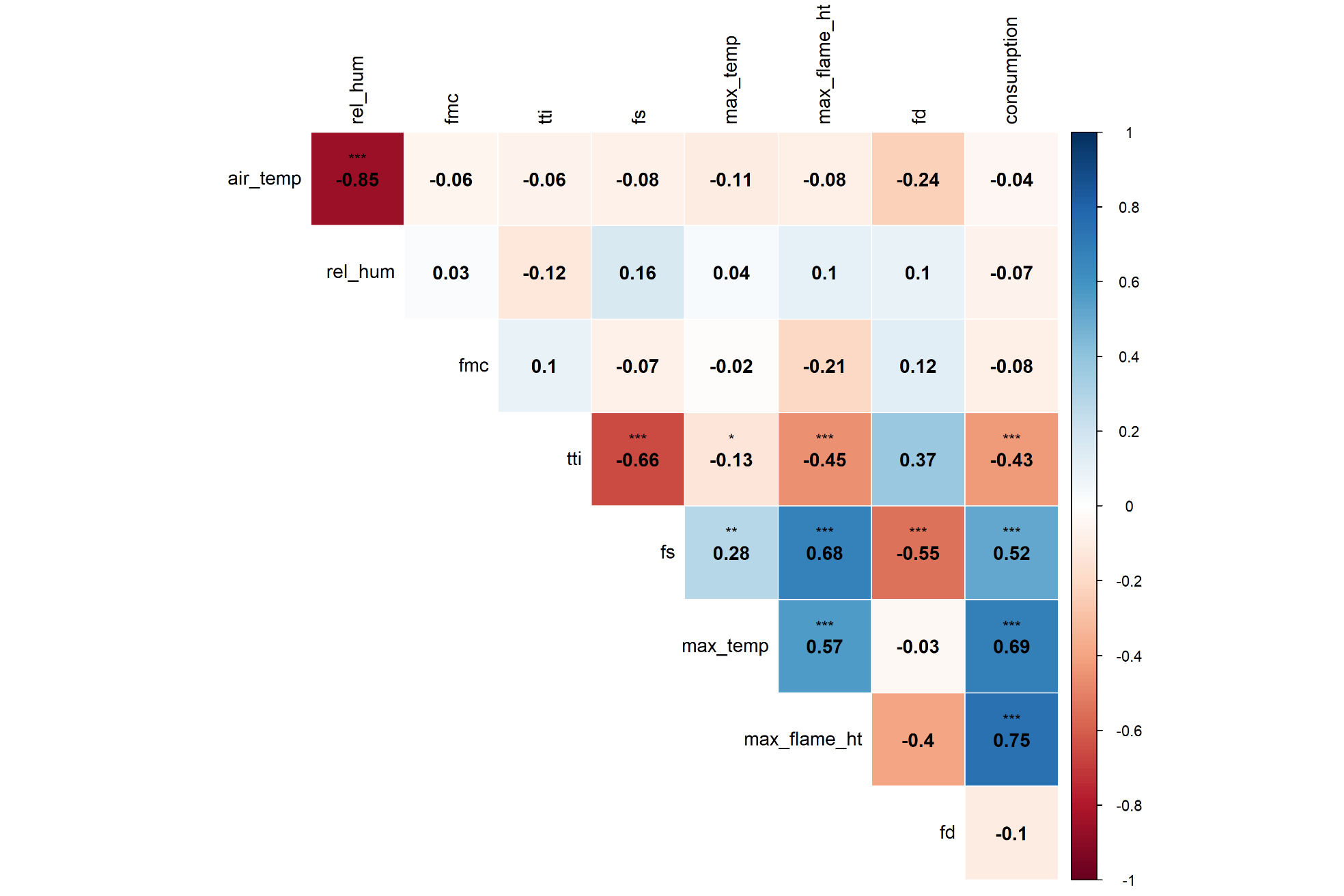
Appendix S2.** Correlation plot among flammability metrics and ambient conditions, table of mean leaf trait values, table of mean litter flammability and correlations between leaf traits and litter flammability.

**Figure S1.** Spearman’s rho correlations between ambient air temperature, relative humidity, fuel moisture content and flammability metrics for the circular tray experiments (both single species and mixed litter bed results, n = 58). Significance values were determined using the Spearman’s rank correlation test: * *P* ≤ 0.05, ** *P* ≤ 0.01 and *** *P* ≤ 0.001. air_temp = ambient air temperature, rel_hum = relative humidity, fmc = fine fuel moisture content, tti = time to ignition, fs = flame spread rate, max_temp = maximum temperature of sample when burning, max_flame_ht = maximum flame height, fd = flaming duration, consumption = proportion consumed.

**Table S1.** Leaf traits for species studied.

| **Species** | **Leaf area, cm^2^** | **SLA, cm^2^/g** | **Phosphorus content, mg/kg** | **Carbon content, %** | **Nitrogen content, %** | **Cellulose, %** | **Lignin, %** | **Total Acid Detergent Fiber, %** | **Ash, %** | **Solubles, %** |
| --- | --- | --- | --- | --- | --- | --- | --- | --- | --- | --- |
| *Ackama paniculosa* | 9.5 | 65.0 |  |  |  |  |  |  |  |  |
| *Callicoma serratifolia* | 15.7 | 96.2 |  |  |  |  |  |  |  |  |
| *Ceratopetalum apetalum* | 23.8 | 73.5 | 401.1 | 44.9 | 0.9 | 22.0 | 23.6 | 45.6 | 0.1 | 54.3 |
| *Cryptocarya foveolata* | 5.9 | 70.8 | 901.9 | 49.9 | 1.4 | 19.0 | 55.5 | 64.5 | 0.1 | 35.4 |
| *Cryptocarya meisneriana* | 12.0 | 113.7 | 664.7 | 47.4 | 1.8 | 21.0 | 32.4 | 50.4 | 0.2 | 49.4 |
| *Doryphora sassafras* | 12.7 | 87.8 | 666.6 | 47.4 | 1.4 | 23.9 | 24.9 | 48.7 | 0.2 | 51.0 |
| *Elaeocarpus reticulatus* | 11.8 | 74.4 |  |  |  |  |  |  |  |  |
| *Eucalyptus campanulata* | 13.7 | 5.5 | 625.0 | 49.7 | 1.5 | 30.1 | 11.5 | 41.6 | 0.3 | 58.1 |
| *Nothofagus moorei* | 14.5 | 64.2 | 603.2 | 48.2 | 1.2 | 19.0 | 48.3 | 61.9 | 0.3 | 37.8 |
| *Orites excelsus* | 23.1 | 96.2 | 366.6 | 44.4 | 0.9 | 21.6 | 24.5 | 46.1 | 0.2 | 53.7 |
| *Persoonia media* | 6.6 | 81.6 | 578.8 | 46.8 | 1.0 | 17.3 | 20.4 | 37.7 | 0.1 | 62.2 |
| *Quintinia verdonii* | 42.9 | 101.2 |  |  |  |  |  |  |  |  |
| *Schizomeria ovata* | 36.3 | 89.2 |  |  |  |  |  |  |  |  |
| *Trochocarpa laurina* | 2.1 | 102.0 |  |  |  |  |  |  |  |  |

**Table S2.** Mean values of litter bed flammability for studied species.

| **Species** | **Time to ignition, s** | **Flame spread rate, cm/s** | **Maximum temperature, °C** | **Maximum flame height, cm** | **Flaming duration, s** | **Consumption, %** |
| --- | --- | --- | --- | --- | --- | --- |
| *Ackama paniculosa* | 48 | 0.11 | 374 | 28 | 134 | 50 |
| *Callicoma serratifolia* | 12 | 0.34 | 683 | 78 | 78 | 95 |
| *Ceratopetalum apetalum* | 20 | 0.20 | 615 | 54 | 113 | 93 |
| *Cryptocarya foveolata* | 57 | NA | 39 | 19 | 43 | 9 |
| *Cryptocarya meisneriana* | 18 | 0.26 | 664 | 61 | 119 | 87 |
| *Doryphora sassafras* | 18 | 0.17 | 453 | 31 | 191 | 74 |
| *Elaeocarpus reticulatus* | 32 | 0.27 | 474 | 50 | 110 | 87 |
| *Eucalyptus campanulata* | 15 | 0.28 | 601 | 71 | 108 | 93 |
| *Nothofagus moorei* | 37 | 0.12 | 608 | 30 | 207 | 75 |
| *Orites excelsus* | 10 | 0.33 | 534 | 58 | 134 | 90 |
| *Persoonia media* | 20 | 0.15 | 417 | 34 | 165 | 38 |
| *Quintinia verdonii* | 9 | 0.35 | 698 | 69 | 106 | 96 |
| *Schizomeria ovata* | 13 | 0.26 | 666 | 39 | 91 | 85 |
| *Trochocarpa laurina* | 26 | 0.19 | 459 | 14 | 153 | 18 |

**Table S3.** Spearman’s rho (*ρ*) correlations between leaf traits and flammability at the litter bed-scale. Correlations shaded are above *ρ* ± 0.70.

| **Trait** | **Time to ignition, s** | **Consumption, %** | **Flaming duration, s** | **Flame spread rate, cm/s** | **Maximum temperature, °C** | **Maximum flame height, cm** |
| --- | --- | --- | --- | --- | --- | --- |
| **Leaf area, cm^2^** | *ρ* = -0.75  *P* = 0.002 | *ρ* = 0.79  *P* = 0.0007 | *ρ* = -0.27  *P* = 0.35 | *ρ* = 0.55  *P* = 0.05 | *ρ* = 0.81  *P* = 0.0004 | *ρ* = 0.64  *P* = 0.01 |
| **SLA, cm^2^/g** | *ρ* = -0.52  *P* = 0.06 | *ρ* = 0.19  *P* = 0.52 | *ρ* = -0.07  *P* = 0.81 | *ρ* = 0.42  *P* = 0.15 | *ρ* = 0.42  *P* = 0.14 | *ρ* = 0.24  *P* = 0.42 |
| **Phosphorus content, mg/kg** | *ρ* = 0.33  *P* = 0.42 | *ρ* = -0.5  *P* = 0.21 | *ρ* = -0.24  *P* = 0.57 | *ρ* = -0.21  *P* = 0.64 | *ρ* = -0.29  *P* = 0.49 | *ρ* = -0.36  *P* = 0.39 |
| **Carbon content, %** | *ρ* = 0.45  *P* = 0.26 | *ρ* = -0.26  *P* = 0.53 | *ρ* = -0.36  *P* = 0.39 | *ρ* = -0.21  *P* = 0.64 | *ρ* = -0.17  *P* = 0.69 | *ρ* = -0.26  *P* = 0.53 |
| **Nitrogen content, %** | *ρ* = -0.02  *P* = 0.96 | *ρ* = -0.02  *P* = 0.96 | *ρ* = -0.19  *P* = 0.65 | *ρ* = -0.04  *P* = 0.94 | *ρ* = 0.19  *P* = 0.65 | *ρ* = 0.21  *P* = 0.61 |
| **Cellulose, %** | *ρ* = -0.73  *P* = 0.04 | *ρ* = 0.71  *P* = 0.05 | *ρ* = -0.23  *P* = 0.59 | *ρ* = 0.54  *P* = 0.22 | *ρ* = 0.3  *P* = 0.47 | *ρ* = 0.54  *P* = 0.17 |
| **Lignin, %** | *ρ* = 0.55  *P* = 0.16 | *ρ* = -0.57  *P* = 0.14 | *ρ* = 0.1  *P* = 0.82 | *ρ* = -0.36  *P* = 0.43 | *ρ* = -0.07  *P* = 0.87 | *ρ* = -0.67  *P* = 0.07 |
| **Total ADF, %** | *ρ* = 0.45  *P* = 0.26 | *ρ* = -0.43  *P* = 0.29 | *ρ* = 0  *P* = 1 | *ρ* = -0.21  *P* = 0.64 | *ρ* = 0  *P* = 1 | *ρ* = -0.57  *P* = 0.14 |
| **Ash, %** | *ρ* = -0.32  *P* = 0.43 | *ρ* = 0.37  *P* = 0.37 | *ρ* = 0.13  *P* = 0.76 | *ρ* = 0.11  *P* = 0.82 | *ρ* = 0.37  *P* = 0.37 | *ρ* = 0.32  *P* = 0.43 |
| **Solubles, %** | *ρ* = -0.45  *P* = 0.26 | *ρ* = 0.43  *P* = 0.29 | *ρ* = 0  *P* = 1 | *ρ* = 0.21  *P* = 0.64 | *ρ* = 0  *P* = 1 | *ρ* = 0.57  *P* = 0.14 |
